# Predicting Nutritional and Morphological Attributes of Fresh Commercial *Opuntia* Cladodes Using Machine Learning and Imaging

**DOI:** 10.3390/jimaging12020067

**Published:** 2026-02-05

**Authors:** Juan Arredondo Valdez, Josué Israel García López, Héctor Flores Breceda, Ajay Kumar, Ricardo David Valdez Cepeda, Alejandro Isabel Luna Maldonado

**Affiliations:** 1 Department of Agricultural and Food Engineering, Faculty of Agronomy, Autonomous University of Nuevo Leon, Francisco Villa S/N, Ex-Hacienda El Canadá, General Escobedo CP 66050, Nuevo León, Mexico; 2 Department of Plant Breeding, Antonio Narro Autonomous Agrarian University, Calzada Antonio Narro 1923, Buenavista, Saltillo CP 25315, Coahuila, Mexico; 3 Department of Biosystems and Agricultural Engineering, Oklahoma State University, Stillwater, OK 74078, USA; 4 Center-North Regional University Center, Autonomous University of Chapingo, Calle Cruz del Sur 100, Col. Constelación, Zacatecas CP 98085, Zacatecas, Mexico

**Keywords:** *Opuntia* commercial cladodes, chlorophylls, plant mineral content, phenolic compounds, morphological traits, image processing, machine learning

## Abstract

*Opuntia ficus-indica* L. is a prominent crop in Mexico, requiring advanced non-destructive technologies for the real-time monitoring and quality control of fresh commercial cladodes. The primary research objective of this study was to develop and validate high-precision mathematical models that correlate hyperspectral signatures (400–1000 nm) with the specific nutritional, morphological, and antioxidant attributes of fresh cladodes (cultivar Villanueva) at their peak commercial maturity. By combining hyperspectral imaging (HSI) with machine learning algorithms, including K-Means clustering for image preprocessing and Partial Least Squares Regression (PLSR) for predictive modeling, this study successfully predicted the concentrations of 10 minerals (N, P, K, Ca, Mg, Fe, B, Mn, Zn, and Cu), chlorophylls (a, b, and Total), and antioxidant capacities (ABTS, FRAP, and DPPH). The innovative nature of this work lies in the simultaneous non-destructive quantification of 17 distinct variables from a single scan, achieving coefficients of determination (R^2^) as high as 0.988 for Phosphorus and Chlorophyll b. The practical applicability of this research provides a viable replacement for time-consuming and destructive laboratory acid digestion, enabling producers to implement automated, high-throughput sorting lines for quality assurance. Furthermore, this study establishes a framework for interdisciplinary collaborations between agricultural engineers, data scientists for algorithm optimization, and food scientists to enhance the functional value chain of *Opuntia* products.

## 1. Introduction

Opuntia species have gained increasing attention due to their remarkable adaptation to climate change, particularly in arid and semi-arid regions affected by water scarcity. These plants exhibit crassulacean acid metabolism (CAM), which significantly reduces water loss during photosynthesis and enhances water-use efficiency, making Opuntia and other CAM plants increasingly important under climate-change-driven water-limited conditions [1,2,3]. In addition, Opuntia produces exotic and non-traditional fruits and cladodes that are rich in bioactive compounds, including polyphenols and betalains, which have been associated with health-promoting effects. Extracts from Opuntia ficus-indica fruits containing high levels of betalains and phenolic compounds have demonstrated significant biological activities related to metabolic health, such as antioxidants, anti-inflammatory, and anti-steatotic effects. Moreover, different O. ficus-indica varieties exhibit distinct profiles of phenolics and betalains, which have been linked to potential lipid-lowering effects in adipocyte models. Metabolomic studies have further revealed elevated concentrations of phenolics, flavonoids, and betalains in red Opuntia fruits, supporting their antioxidant and antidiabetic potential. Overall, Opuntia species are widely recognized for the abundance of phenolic acids, antioxidants, and betalain pigments in both fruits and cladodes, which collectively contribute to their beneficial health-related properties [4,5,6,7,8,9]. Previous studies have reported that Opuntia cladodes are an important source of essential minerals such as potassium (K), sodium (Na), calcium (Ca), magnesium (Mg), and iron (Fe), with cladodes showing major levels of Ca and K and measurable amounts of Mg, Na, and Fe in their mineral profiles (see review in International Journal of Food Science and Technology and mineral analyses of O. ficus-indica and wild Opuntia species) [10,11,12,13]. The antioxidant capacity of plant polyphenols has been widely documented, highlighting their role in protecting against oxidative stress [14,15,16]. In this context, El-Guezzane et al. reported high antioxidant activity and phenolic content in Opuntia ficus-indica cultivars compared to other species [17].

Plant responses to abiotic stress involve complex physiological and metabolic adjustments. Salinity stress, for instance, has been shown to induce the accumulation of proteins and proline as adaptive resistance mechanisms [18], while metabolic regulation plays a key role in maintaining cellular osmotic balance under adverse environmental conditions [19]. Optical properties of plant tissues are closely linked to these physiological changes. Leaf and cladode spectral reflectance varies according to plant species, tissue water content, and structural characteristics [20]. Moreover, morphological and colorimetric descriptors have proven useful for characterizing *Opuntia* tissues and assessing their physiological status [21].

In green vegetation, the near-infrared (NIR) region of the electromagnetic spectrum (approximately 690–740 nm) is particularly relevant, as chlorophyll strongly absorbs radiation below 700 nm, resulting in low reflectance, while reflectance increases sharply beyond this threshold [22]. Hyperspectral imaging (HSI) has therefore emerged as a powerful non-destructive tool for plant phenotyping. Several studies have demonstrated the potential of HSI for estimating photosynthetic and biochemical traits, including chlorophyll content, in different crops such as maize and rice [23,24]. According to Benelli et al., HSI enables high-throughput assessment of plant physiology, fruit maturity, and biotic stress, supporting precision agriculture applications [25].

Despite its advantages, HSI generates high-dimensional and noisy datasets, requiring effective preprocessing and dimensionality reduction techniques. Principal Component Analysis (PCA) is commonly employed to reduce data redundancy by transforming correlated spectral variables into a smaller set of uncorrelated principal components that explain most of the variance [26]. Prior to PCA, spectral preprocessing methods are commonly applied to improve data quality by reducing instrumental noise and correcting physical variations. Techniques such as Savitzky–Golay (SG) smoothing are frequently used to reduce noise, while scatter correction methods like Standard Normal Variate (SNV) and Multiplicative Scatter Correction (MSC) normalize spectra to minimize baseline shifts and multiplicative light-scattering effects. These preprocessing steps are often applied in combination before multivariate analyses, including PCA, to ensure accurate and reliable chemometric results [27,28]. In addition, hyperspectral indices such as the normalized vegetation difference index (NDVI) and its variants, including vNDVI, have been proposed to enhance vegetation monitoring accuracy [29].

For image segmentation and spectral preprocessing, unsupervised machine learning algorithms such as K-Means clustering are widely used due to their computational efficiency and their ability to group pixels with similar spectral characteristics. K-Means is considered a standard unsupervised classification algorithm in remote sensing and image analysis, efficiently partitioning large datasets into clusters of spectrally similar pixels. This method is broadly implemented in image processing applications to segment images by grouping similar pixels or regions into the same classes, highlighting its versatility and practical utility in both spectral and spatial analyses [30,31,32,33]. K-Means clustering algorithm has many uses, including grouping text documents, images, and videos [34]. Unlike deep learning-based approaches, which require large, labeled datasets, K-Means leverages spectral similarity and spatial continuity, making it particularly suitable for complex plant tissues such as succulent cladodes. Since multivariate analysis is needed for HSI analysis, Dao et al. demonstrated the usefulness of integrating deep learning and hyperspectral data in studies related to drought in pastures [35]. Izzo et al. found that hyperspectral curves frequently exhibit correlated predictor variables, causing multicollinearity, given the contiguous nature of spectral data [36].

The contiguous nature of hyperspectral data often leads to multicollinearity among predictor variables, while the number of spectral bands typically exceeds the number of samples. To address these challenges, Partial Least Squares Regression (PLSR) is frequently applied in multivariate analysis because it simultaneously reduces the dimensionality of the predictor space while maximizing the covariance between predictors and response variables [37,38]. By constructing latent components that capture the greatest covariance between linear combinations of predictors (X) and response variables (Y), PLSR effectively handles datasets with many correlated predictors or collinearity [39]. To use PLSR for assessing model performance, it is recommended to split the entire dataset into one for model training and one for out-of-sample validation to provide a more robust and accurate assessment of the prediction [40]. This makes it particularly suitable for applications in chemometrics, spectroscopy, and other fields requiring reliable predictive modeling while maintaining interpretability of the underlying relationships [41,42]. To use PLSR for assessing model performance, it is recommended to split the entire dataset into one for model training and one for out-of-sample validation to provide a more robust and accurate assessment of the prediction. Value Inflation Factor (VIF) values in multiple linear regression models further justify the use of PLSR for robust multivariate modeling [43]. Best practices recommend dividing datasets into training and validation subsets to ensure reliable model performance assessment.

Although machine learning and hyperspectral techniques have been extensively applied in agri-food research, their combined use for the simultaneous prediction of mineral nutrients, chlorophylls, and antioxidant compounds in fresh *Opuntia ficus-indica* cladodes remains limited. Therefore, the aim of this study was to develop non-destructive, high-precision mathematical models correlating hyperspectral images (400–1000 nm) with the mineral, chlorophyll, and antioxidant contents of fresh commercial cladodes. Additionally, this study evaluates the effectiveness of K-Means clustering as a preprocessing strategy to enhance signal-to-noise ratio, extracts key morphological descriptors using digital image processing, and proposes an alternative to conventional laboratory acid digestion methods for industrial quality control in the *Opuntia* food chain.

## 2. Materials and Methods

### 2.1. Plant Material and Sample Preparation

The study was conducted using 15 fresh cladodes of *Opuntia ficus-indica* (L.) Mill. (cv. Villanueva). The samples were obtained from the experimental field of the Faculty of Agronomy at the Autonomous University of Nuevo Leon, Mexico. Only third-level cladodes (third generation from the main trunk) were selected to ensure uniformity of physiological maturity. This developmental stage corresponds to the commercial maturity commonly used for nopalitos (cladodes) intended for human consumption.

### 2.2. Sampling and Firmness of Fresh Cladodes

Fresh cladodes were harvested from the third level of two-year-old *Opuntia ficus-indica* plants grown in pots containing unfertilized clay loam soil. The samples were weighed using a precision balance and photographed with an iPhone 11 smartphone. The images were analyzed using ImageJ software (version 2022; National Institutes of Health, Bethesda, MD, USA) to obtain morphological descriptors.

Cladode firmness was subsequently measured using a texture analyzer (XT Plus Texture Analyzer, Stable Micro Systems, Godalming, UK). Three puncture tests were performed on each cladode using a flat probe with a diameter of 1 mm. The penetration speed was set at 2 mm·s^−1^. Firmness was expressed as the maximum force (N) recorded during the puncture test.

### 2.3. Hyperspectral Image Acquisition

Hyperspectral images (HSI) were captured using a laboratory-scale push-broom hyperspectral imaging system. The system consists of a high-resolution camera operating in the visible and near-infrared (VNIR) range of 400 to 1000 nm. Cladodes were placed on a motorized translation stage with controlled lighting to ensure uniform reflectance. To calibrate the system and account for environmental lighting variations, a white reference (99% reflectance) and a dark reference (0% reflectance) were acquired before each scanning session.

### 2.4. Image Data Processing and K-Means Clustering

Raw hyperspectral data (hypercubes) were processed to extract meaningful spectral signatures, and the segmentation of the cladode from the background was performed using K-Means clustering. This unsupervised machine learning algorithm was chosen over deep learning or superpixel methods due to its computational efficiency in handling high-dimensional data without requiring extensive manual labeling. In succulent tissues such as *Opuntia*, K-Means effectively groups pixels with similar spatial-spectral characteristics, enabling the isolation of representative tissue regions while minimizing noise from surface reflections or background interference.

### 2.5. Spectral Preprocessing and Feature Extraction

Before image acquisition, the hyperspectral imaging system was powered on to achieve thermal and temporal stability [44]. Images were captured at 30 frames per second (fps) with an exposure time of 20 ms. A conveyor speed of 7 mm/s was used to optimize the frame aspect ratio. Hyperspectral images of fresh cladodes were acquired with dimensions 1024 × 768 × 600, where the first two dimensions represent spatial coordinates and the third represents spectral values, forming a hypercube. Hypercube construction and initial processing were performed in Matlab R2021a.

Spectral data were preprocessed to enhance spectral features and reduce the number of spectra using the Standard Normal Variate (SNV) technique. Color clustering of the fresh cladode images was then performed with K-Means using the HiperTools v3 software [45]. Given the multidimensional nature of hyperspectral data, K-Means provides a computationally efficient alternative to methods like hierarchical clustering [46,47]. Noise was quantified using the signal-to-noise ratio (*SNR*), calculated as:

(1) $$SNR=\frac{\sigma_{signal}^{2}}{\sigma_{noise}^{2}}$$

A high *SNR* (≫1) indicates precise data, while a low *SNR* indicates noisy measurements. The dimensions of interest were assumed to correspond to directions of highest variance and *SNR*. To further improve SNR, signal clustering with K-Means has been shown to identify optimal image regions by leveraging spatial relationships, as neighboring pixels often exhibit similar signals [48].

Selecting the number of clusters (K) is critical for K-Means performance. Two commonly used methods are the Elbow Method and Silhouette Analysis. The Elbow Method identifies the optimal K by plotting cluster distortion versus K. As K increases, the average distance of points from cluster centroids decreases, and the “elbow” point, where distortion reduction slows, is chosen as the optimal K [49]. Silhouette Analysis evaluates cluster separation by measuring how similar a point is to its own cluster compared with neighboring clusters. Silhouette coefficients range from −1 to 1: values near +1 indicate well-separated clusters, 0 indicates proximity to the cluster boundary, and negative values suggest misassigned points [50].

Finally, a 2nd derivative Savitzky–Golay smoothing was applied using HyperTools 3.0 to further reduce noise and enhance spectral feature resolution.

### 2.6. Laboratory Reference Analysis

All *Opuntia* cladodes samples were analyzed in triplicates. Samples were weighed and dried in a Yamato DX 602C oven (Yamato Scientific Co., Ltd., Tokyo, Japan) at 60 °C for 72 h and subsequently pulverized with a mortar, separating 10 g of cladode flour. The flour was then subjected to acid digestion in a mixture of perchloric acid and nitric acid [51]. Nitrogen (N) was quantified by the micro Kjendahl method according to Bremner [52]. The contents (mg/100 g) of phosphorous (P), potassium (K), calcium (Ca), magnesium (Mg), iron (Fe), boron (B), manganese (Mn), zinc (Zn) and copper (Cu) were determined by acid digestion extract using a coupled plasma induction atomic emission spectrometer (ICP-AES, Agilent 725-ES, Agilent Technologies, Santa Clara, CA, USA).

Chlorophylls were obtained using method described by Delgado-Vargas et al. [53]. First, 25 mL of 80% methanol was added to 0.5 g of pulp sample and homogenized, then centrifuged at 5000 *g* for 5 min at 4 °C, and the absorbance values at 663, 646 and 470 nm were obtained. The chlorophyll was calculated using Equations (2)–(4).

Chloropyll a: Ca = 12.25A663 − 2.29A646(2)

Chloropyll b: Cb = 21.5A645 − 5.21A663(3)

Chloropyll total: C(a + b) = 7.15A663 + 18.71A470(4)

To determine the antioxidant capacity, the ABTS method was used as proposed by Rice-Evans et al. and modified by Ozgen et al. for FRAP and DPPH [54,55].

### 2.7. Model Development and Statistical Validation

Hyperspectral data (acquisition and extraction of spatial and spectral information) were obtained from images of fresh cladodes of *Opuntia*. Once the arrangements of the hyperspectral signature matrix and the K-means centroids were obtained, the PLSR algorithm was applied with cross-validation to find the optimal number of components and then they were used in the PLSR algorithm for analysis, according to Equation (5).

(5) Y=XW_h [(P_h^′ W_h)]^(−1) C_h^′+ε_h

where *Y* is the matrix of dependent variables, *X* is the matrix of explanatory variables; *W_h* is the weight matrix of X, generated by the algorithm; *P_h*^′ is the charge matrix, transposed; *C_h*^′ is the weight matrix of Y, transposed; *ε_h* is the residual matrix. The subscript h of Equation (1) indicates the number of components to be used.

For the PLSR processing used for all the reflectance values of the images of fresh *Opuntia* cladodes, a cross-validation was performed to optimize the number of components, using *h* = 9 for the minerals and *h* = 5 for the rest of the variables. Finally, a partial least squares regression (PLSR) statistical analysis was performed with Minitab v2021 [56].

## 3. Results

The harvested fresh cladodes had an average weight of 103 g and a mean penetration resistance of 20.6 N (2.10 kg). The mineral composition of the fresh cladodes is summarized in Table 1, highlighting the concentrations of macro- and micronutrients (ppm), as well as chlorophyll and antioxidant contents (mg·100 g^−1^).

The images of the fresh cladodes, once processed with ImageJ, provided the mean morphological values of the cladodes, which are shown in Table 2. ImageJ has subtraction routines to remove the background of the image and can calculate the area, statistics pixel values, distances, and angles from user-defined selections, as well as create density histograms and line profile plots [57]. The following morphological characteristics of *Opuntia* commercial cladodes were obtained with ImageJ: mean area of 212 cm^2^, perimeter of 62.1 cm and lengths Lmax and Lmin with values that ranged between 21.2 and 13.3 cm, respectively (Table 2).

PLSR analysis was performed using the reflectance intensity values, the matrix (15 × 768) as the independent variable. K-Means clustering of the fresh cladodes image was performed. A general model for the use of PLSR is shown by Equation (6).

[M_((15 × 17))] = β_oi + [λ_((15 × 768))] [β_((768 × 17))] + ε_((15 × 17))(6)

where M is the matrix of 10 minerals and 7 metabolites from 15 cladodes, which is the dependent variable; β_oi the constant coefficients; λ the 768-pixel matrix of reflectance intensity of 15 cladodes, the independent variables; β are the values of the matrix of 768 coefficients for the 17 variables and ε is the error.

From the analysis of variance (Table 3), the values of the mean square error, the coefficient of determination (R^2^) and the level of statistical significance for the mineral, chlorophylls and antioxidants contents were obtained.

**Figure 1:**
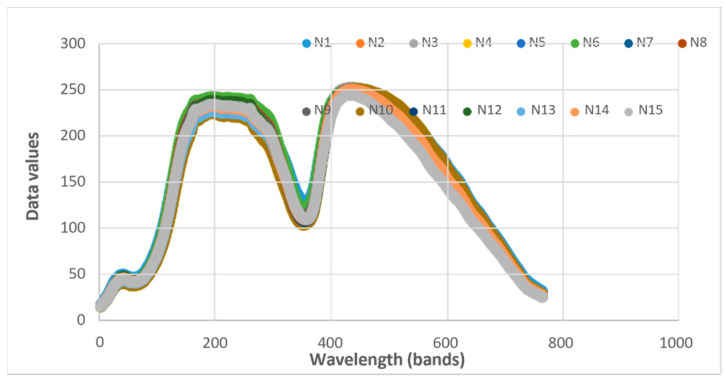
Data values of the K-Means spectra of 15 cladodes in 768 pixels.

Figure 1 displays the K-Means spectral profiles for 15 cladodes across 768 bands. The results demonstrate high intra-class stability, with characteristic reflectance peaks near data value 250. The prominent absorption feature at band 350 and the subsequent decay in the higher bands are indicative of the specific succulent morphology and water-rich composition of the cactus pads.

The contents of mineral nutrients, chlorophylls, and antioxidants versus hyperspectral images showed high coefficients of determination, good levels of significance and a variance of 0.99 (Table 3). After the analysis of the individual tested compounds with PLSR resulted in lower R^2^ values obtaining a better behavior with all the dependent variables at the same time, by generating a greater number of data, for λ (15 × 768) of all the pixels versus all the compounds, obtaining the order of R^2^ = 0.98 for P, and for other minerals, until obtaining the lowest value of R^2^ = 0.659 for DPPH.

The values ranging from 0.7 to 0.871 were obtained from the PLSR analysis as explanatory percentages and significant values for the variables, with 95% confidence.

**Figure 2:**
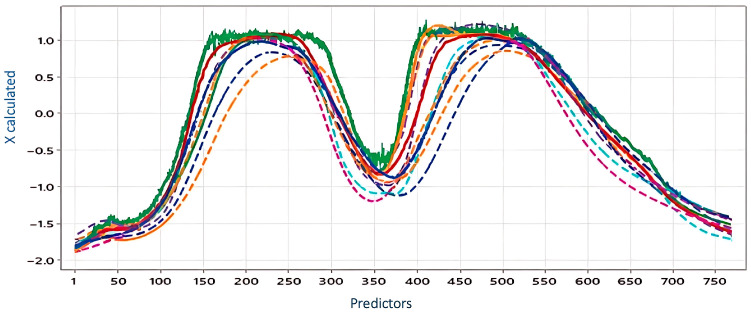
Predicted values of the reflection intensities of the fresh cladode.

Figure 1 shows the wavelengths (λ) computed by evaluating pixels. The R^2^ values were around 0.98 for Mn, to 0.99 for the others and showed the variation in the mineral contents from 0.70 in the phenols, up to 0.87, indicating that the mineral contents significantly affect the reflectance. From the PLSR analysis, the estimated wavelengths were obtained and are shown in Figure 2.

To assess the relationship between spectral data and chemical composition, PLSR coefficients were calculated for mineral nutrients, chlorophyll pigments, and antioxidant capacity across the 768 spectral bands. Figure 2 illustrates the predicted reflection intensities for the fresh cladode samples.

**Figure 3:**
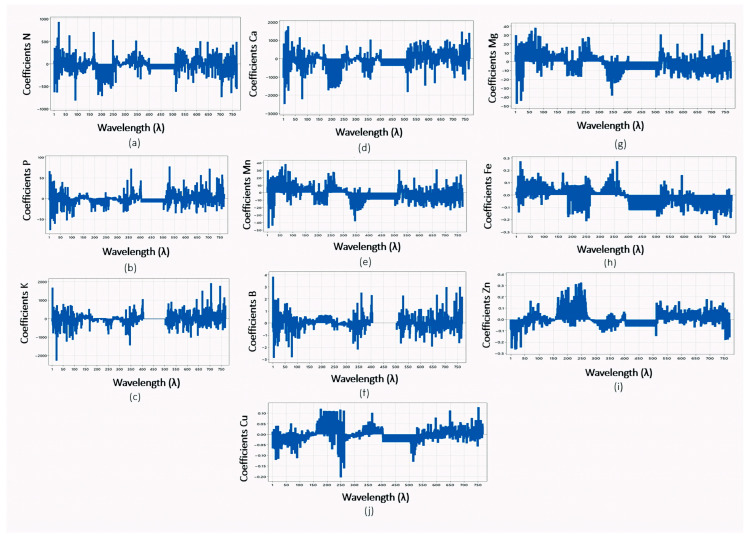
PLSR coefficients for N (**a**), P (**b**), K (**c**), Ca (**d**), Mn (**e**), B (**f**) Mg (**g**), Fe (**h**), Zn (**i**) and Cu (**j**) models (9 components were selected).

The distribution of the regression coefficients, which identify the spectral regions most influential to each model, is presented in Figure 3 for macro and micronutrients (N, P, K, Ca, Mn, B, Mg, Fe, Zn, and Cu).

**Figure 4:**
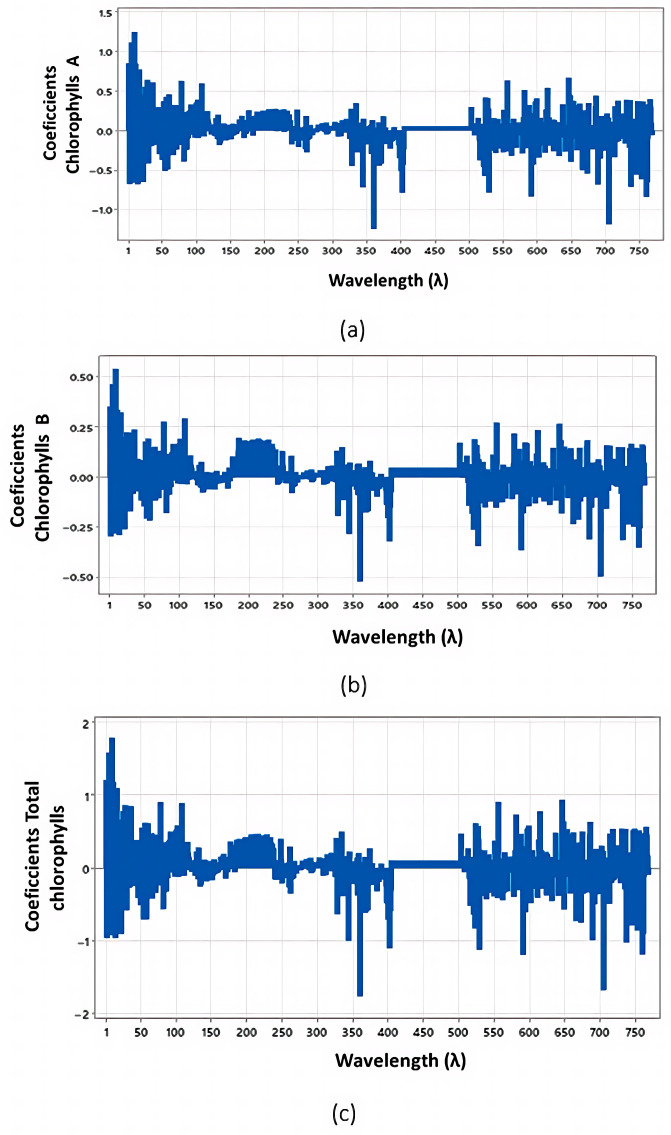
PLSR coefficients for a (**a**), b (**b**) and total chlorophylls (**c**).

**Figure 5:**
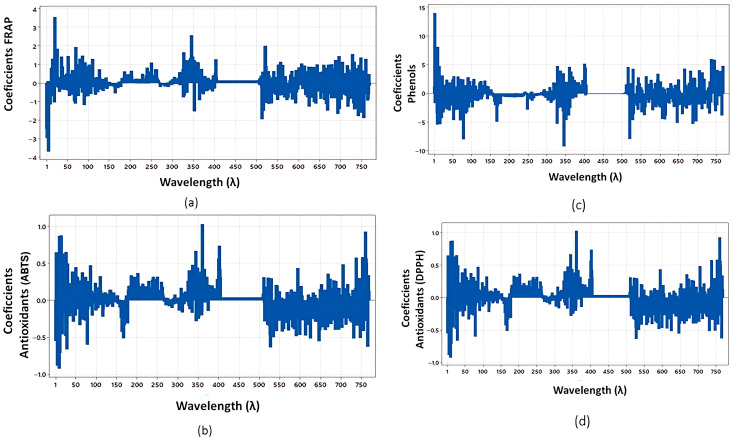
PLSR Coefficients for ABTS (**a**), FRAP (**b**), Phenols (**c**) and Antioxidants (DPPH) (**d**).

Furthermore, the coefficient profiles for chlorophyll a, b, and total chlorophyll are detailed in Figure 4, while antioxidant activities (FRAP, ABTS, Phenols, and DPPH) are shown in Figure 5. For all models, 9 principal components were selected to optimize predictive performance.

The applied methodology can be used to obtain prediction models between other agronomic characteristics or between chemical or biochemical compounds of plants and their hyperspectral images.

The comparative analysis between the experimentally measured values and the PLSR-predicted concentrations for minerals, chlorophylls, and antioxidants is summarized in Table 4. The results demonstrate an exceptional level of predictive accuracy across all parameters, with predicted values closely mirroring the measured laboratory data. For instance, the Nitrogen (N) model yielded a predicted mean of 16,714.44 ppm compared to the measured 16,718.33 ppm, showing minimal deviation. Similar high-precision correlations were observed for macrominerals including Calcium (Ca) and Potassium (K), as well as for bioactive compounds, including Total Chlorophyll and antioxidant capacity (DPPH and FRAP). This near-identity between measured and calculated means confirms the robustness of the 9-component PLSR models in non-destructively estimating the chemical composition of fresh cladodes.

## 4. Discussion

The mineral nutrient content observed in fresh cladodes was consistent with control values reported by Zúñiga-Tarango et al. and Santiago-Lorenzo et al. [58,59], as expected since no external fertilization was applied. In contrast, significant differences emerged when comparing our results with those of Mayer and Cushman, Mokoboki and Sebola, and Nobel [60,61]. These discrepancies are mainly due to ontogenetic and environmental factors, as previous studies analyzed mature cladodes for cattle feed, whereas this study focused on fresh tissue for human consumption. Differences in sampling periods and cultivars further explain variations in macro- and micronutrient concentrations [62,63].

For predictive modeling, the selection of the PLSR algorithm was critical to achieving high accuracy in estimating chemical and biochemical compounds. The PLS1 approach was implemented using the NIPALS (Nonlinear Iterative Partial Least Squares) algorithm, which, unlike PCR or PLS2, computes independent score and loading vectors for each response variable, enabling more precise individual predictions [64]. Model optimization was performed through cross-validation, resulting in nine components that maximize covariance between predictors (spectral bands) and response variables while preventing overfitting in datasets with highly correlated predictors [65].

Regression coefficients provided a robust tool for variable selection by quantifying the influence and direction of specific wavelengths on model performance [66]. The close agreement between laboratory measurements and PLSR predictions, supported by high R^2^ values (Table 4), confirms that hyperspectral imaging combined with PLSR is a reliable non-destructive predictive technique. This approach can be effectively extended to predict additional agronomic and biochemical traits in cactus pear and related species.

The primary innovation of this study lies in the development of a limited-interference predictive framework capable of extracting 17 variables from a single non-destructive scan. Unlike previous studies that relied on colorimetric methods, this research employs K-means clustering to maximize the signal-to-noise ratio in succulent tissues, which are traditionally challenging to analyze due to high water content. Strong predictive performance for minerals suggests that mineral–water complexes significantly influence reflectance in the near-infrared (NIR) region. Slightly lower accuracy for antioxidant capacity (DPPH) may be due to dominant chlorophyll absorption peaks near 700 nm, which partially mask more subtle antioxidant spectral signatures.

Building on previous work on morphological descriptors [20], this study bridges the gap between external physical traits and internal chemical composition. Incorporating recent literature situates the findings within a modern statistical framework, moving beyond outdated methodological justifications.

Despite the high predictive accuracy achieved, limitations exist. The use of 15 third-level cladodes from the Villanueva cultivar ensured high intra-class stability for model calibration but does not fully capture the genetic diversity of the species. Models were developed under greenhouse-controlled conditions, and their performance in open-field environments remains untested. Future research will expand the dataset to multiple cultivars and growing conditions, explore deep learning–based segmentation, and evaluate seasonal and soil-type effects to improve model robustness and generalizability.

## 5. Conclusions

The use of hyperspectral imaging (HSI) supported by partial least squares regression (PLSR) proved to be a reliable approach for modeling mineral nutrients, chlorophylls, and antioxidant contents from hyperspectral images. Although the individual analysis of compounds using PLSR yielded moderate coefficients of determination (R^2^ ranging from 0.70 to 0.87, with statistically significant variables at a 95% confidence level), a strong agreement was observed between the original laboratory averages and the predicted means. Some discrepancies were detected in the estimation of macro- and micro-mineral nutrients; however, the models showed high reliability for key elements such as phosphorus and potassium.

Overall, this research demonstrates that hyperspectral imaging combined with machine learning represents a transformative tool for the *Opuntia* industry, enabling the replacement of traditional, time-consuming chemical analyses with accurate, non-destructive alternatives. The practical significance of this work lies in its applicability to real-time field phenotyping and automated industrial sorting, where integration into conveyor systems could allow instantaneous assessment of harvest maturity and nutrient density. Furthermore, the study lays the groundwork for interdisciplinary collaboration between agricultural engineers and data scientists, supporting the transition of the *Opuntia* value chain toward high-throughput precision agriculture.

## Figures and Tables

**Table 1:** Nutrients found in fresh cladodes.

Component	Unit	Value
Macronutrients		
N	ppm	1670
P	ppm	1600
K	ppm	16,500
Ca	ppm	31,300
Mg	ppm	3370
Micronutrients		
Iron (Fe)	ppm	31.65
Copper (Cu)	ppm	4.71
Zinc (Zn)	ppm	10.49
Manganese (Mn)	ppm	22.15
Boron (B)	ppm	41.71
Bioactive compounds		
Total phenols	mg GAE·100 g^−1^	56.21
Antioxidant activity (DPPH)	mg TE·100 g^−1^	5.69
Antioxidant activity (ABTS)	mg TE·100 g^−1^	31.90
Antioxidant activity (FRAP)	mg TE·100 g^−1^	23.03
Chlorophyll content		
Chlorophyll a	mg·100 g^−1^	5.41
Chlorophyll b	mg·100 g^−1^	2.17
Total chlorophyll	mg·100 g^−1^	7.58

**Table 2:** Fresh cladodes morphological descriptors obtained by using ImageJ.

Area (cm^2^)	Perimeter (mm)	Circularity	Aspect Ratio
212.19	621.29	0.78	1.62
Roundness	Solidity	Lmáx (mm)	Lmin (mm)
0.62	0.98	211.86	133.74

Area, surface area of the cladode (cm^2^); Perimeter, boundary length (mm); Circularity, shape descriptor where 1.0 indicates a perfect circle and values < 1 indicate deviation from circularity; Aspect ratio, ratio of the major axis to the minor axis of the cladode; Roundness, measure of how circular the shape is; Solidity, ratio of the object area to its convex hull area, indicating smoothness of edges; Lmax, maximum Feret diameter (mm); Lmin, minimum Feret diameter (mm).

**Table 3:** PLSR models for nutrients, chlorophylls a (Cl_a), b (Cl_b), total (Cl_T) and antioxidants.

Chemistry Elements or Compounds	F Value	*p* Value	Variance	RMSE	R^2^
N	5.3	0.04 *	0.871	105.12	0.905
K	12.45	0.00 **	0.871	189.04	0.957
P	49.23	0.04 **	0.871	5.667	0.988
Ca	5.56	0.04 **	0.871	230.13	0.909
Mg	7.92	0.02 **	0.871	40.919	0.934
Fe	8.4	0.01 **	0.871	0.306	0.937
Cu	11.16	0.01 **	0.871	0.059	0.952
Zn	16.62	0.00 **	0.871	0.099	0.967
Mn	12.45	0.01 **	0.871	0.453	0.957
B	10.02	0.01 **	0.871	0.489	0.947
Cl_a	46.16	0.00 **	0.715	0.377	0.962
Cl_b	158.23	0.00 **	0.715	0.086	0.988
Cl_T	127.67	0.00 **	0.715	0.317	0.986
Phenols	26.84	0.00 **	0.702	4.020	0.937
DPPH	3.48	0.050 *	0.702	1.753	0.659
ABTS	5.99	0.010 **	0.702	5.328	0.769
FRAP	28.01	0.00 **	0.702	1.484	0.937

* indicates statistical significance at *p* < 0.05, and ** indicates statistical significance at *p* < 0.01.

**Table 4:** Mean values of the contents of minerals, chlorophylls, and antioxidants in ppm, measured, and calculated (predicted) using the PLSR model.

Nm_ Predicted	Nm_ Measured	Pm_ Predicted	Pm_ Measured
16,714.439	16,718.333	1618.384	1618.348
Km_ predicted	Km_ measured	Cam_ predicted	Cam_ measured
16,551.915	16,546.233	31,387.757	31,382.223
Mgm_ predicted	Mgm_ measured	Fem_ predicted	Fem_ measured
3370.852	3370.651	31.648	31.645
Cum_ predicted	Cum_ measured	Znm_ predicted	Znm_ measured
4.712	4.713	10.490	10.490
Mnm_ predicted	Mnm_ measured	Bm_ predicted	Bm_ measured
22.144	22.147	41.708	41.706
Phenolsm_ predicted	Phenolsm_ measured	AntioxDPPHm_ predicted	AntioxDPPHm_ measured
56.160	56.206	5.686	5.686
AntioxABTSm_ predicted	AntioxABTSm_ medido	AntioxFRAPm_ predict	AntioxFRAPm_ measured
31.899	31.896	23.026	23.026
Chloropyll am_ predicted	Chloropyll am_ measu	Chloropyll bm_ predict	Chloropyll bm_ measured
5.411	5.411	2.170	2.169
Chloropyll Tm_ predicted	7.581	Chloropyll Tm_ measured	7.581

## Data Availability

The raw data supporting the conclusions of this article will be made available by the authors on request.
